# Kinetics of Oncolytic Reovirus T3D Replication and Growth Pattern in Mesenchymal Stem Cells 

**DOI:** 10.22074/cellj.2020.6686

**Published:** 2019-12-15

**Authors:** Razieh Sadat Banijamali, Hoorieh Soleimanjahi, Sara Soudi, Hesam Karimi, Asghar Abdoli, Seyed Mahmood Seyed Khorrami, Keivan Zandi

**Affiliations:** 1. Department of Virology, Faculty of Medical Sciences, Tarbiat Modares University, Tehran, Iran; 2. Department of Immunology, Faculty of Medical Sciences, Tarbiat Modares University, Tehran, Iran; 3. Department of Hepatitis and AIDS, Pasteur Institute of Iran, Tehran, Iran; 4. Center for AIDS Research, Laboratory of Biochemical Pharmacology, Department of Pediatrics, Emory University School of Medicine, Atlanta, Georgia, USA

**Keywords:** Cancer, Mesenchymal Stem Cells, Oncolytic Viruses, Quantitative Real-Time Polymerase Chain Reaction, Reovirus Type 3

## Abstract

**Objective:**

Currently, application of oncolytic-virus in cancer treatment of clinical trials are growing. Oncolytic-reovirus is an attractive anti-cancer therapeutic agent for clinical testing. Many studies used mesenchymal stem cells (MSCs) as a carrier cell to enhance the delivery and quality of treatment with oncolytic-virotherapy. But, biosynthetic capacity and behavior of cells in response to viral infections are different. The infecting process of reoviruses takes from two-hours to one-week, depends on host cell and the duration of different stages of virus replication cycle. The latter includes the binding of virus particle, entry, uncoating, assembly and release of progeny-viruses. We evaluated the timing and infection cycle of reovirus type-3 strain Dearing (T3D), using one-step replication experiment by molecular and conventional methods in MSCs and L929 cell as control.

**Materials and Methods:**

In this experimental study, L929 and adipose-derived MSCs were infected with different multiplicities of infection (MOI) of reovirus T3D. At different time points, the quantity of progeny viruses has been measured using virus titration assay and quantitative real-time polymerase chain reaction (qRT-PCR) to investigate the ability of these cells to support the reovirus replication. One-step growth cycle were examined by 50% cell culture infectious dose (CCID_50_) and qRT-PCR.

**Results:**

The growth curve of reovirus in cells shows that MOI: 1 might be optimal for virus production compared to higher and lower MOIs. The maximum quantity of virus production using MOI: 1 was achieved at 48-hours post-infection. The infectious virus titer became stationary at 72-hours post-infection and then gradually decreased. The virus cytopathic effect was obvious in MSCs and this cells were susceptible to reovirus infection and support the virus replication.

**Conclusion:**

Our data highlights the timing schedule for reovirus replication, kinetics models and burst size. Further investigation is recommended to better understanding of the challenges and opportunities, for using MSCs loaded with reovirus in cancer-therapy.

## Introduction

Oncolytic viruses (OVs) have emerged as an efficient and promising new class of therapeutic agents to combat cancers and started a new era in cancer therapy (1). Recently, the clinical trials showed the effectiveness of OVs in human cancers. The US Food and Drug Administration (FDA) approved herpes virus based-OV for the treatment of progressive metastatic melanoma (2). Currently, there are a large number of other OVs under investigation in clinical trials (1).

Reovirus is a naturally occurring OV that has been used in therapy for a broad spectrum of human cancers (3). Many clinical trials evaluated the potential application of an oncolytic reovirus developed by Reolysin®, (pelareorep; wild-type reovirus; Serotype 3 Dearing; Oncolytics Biotech Inc.), for the treatment of different tumor cells (4). In 2015, the FDA has approved Reolysin®, as a first-in-class systemically administered an attractive anti-cancer agent for malignant glioma, ovarian and pancreatic cancers (2). The reovirus ability to selective replication in cancer cells is due to cancer cells mutations on a growth pathway known as the RAS signaling pathway (5). Reovirus considered relatively benign, but targets the gastrointestinal and upper respiratory tracts in newborns and immunocompromised individuals. Reovirus effectively infect and kill many types of transformed cells. Several studies have revealed that the reovirus T3D has oncolytic potential (6, 7). Due to extensive pre-clinical and clinical efficacy, replication competency, and low toxicity profile in humans, reovirus have considered as an attractive anti-cancer therapy in oncolytic virotherapy (8).

Despite the benefits of OVs, the therapeutic efficacy of OVs have been limited due to numerous biological, immunological, physiological and intra tumoral barriers (9). Delivery of the OVs to target sites is one of the major obstacles due to virus elimination by the host antibodies and other immune cells before they reach destination. Several methods have been proposed to evade this particular problem (10). Early experiments showed the enhanced antitumor activity of virus-producing cells compared with naked viruses (11). Recent approaches tried to combine OVs with other methods like “smarter” carrier to improve delivery of the OVs (12). This finding led to the hypothesis that carrier cells could be used to hide the therapeutic virus from the host immune system and guarantee the biologically active virus transferring toward the target site (11). Several preclinical studies have extensively evaluated many different cell as carriers for oncolytic virotherapy (10). The viruses can be loaded onto cells without losing the biological activity of either virus or cell carrier (13).

In recent years, mesenchymal stem cells (MSCs) have received significant attention as efficient vehicle to transfer OVs towards the cancer cells (10, 14). MSCs known as fibroblast-like non-hematopoietic stem cells have been isolated from bone marrow (BM), adipose, fetal liver, placenta and umbilical cord blood. These cells are positive for surface markers CD105, CD73, and CD90 and lack expression of endothelial and hematopoietic lineage markers including CD45, CD34, CD14 or CD11b, CD79a or CD19, and HLA-DR (15, 16).

Since the cell and virus biology is affected by each other, the study of these changes are necessary to improve their consequences. The aim of the current study was to compare the reovirus growth life cycles and intracellular kinetic models in adipose-derived MSCs (AD-MSCs) as carrier for wild-type oncolytic activity of virus with L929 cell as control cell. For this purpose, monitoring of growth kinetics in one-step growth assays in both cells was investigated. A detailed growth kinetic models and virus production profile was optimized to the best-fit in vitro parameters. It could provide a starting point toward understanding of the virus growth dynamics, propagation and release. Here, we demonstrate a molecular and conventional methods to measure the kinetics of reovirus production in different cells.

## Materials and Methods

### Isolation and culture of adipose-derived-mesenchymal stem cells

In this experimental study, adipose tissue was obtained from six-weeks-old female C57BL/6 mice (Pasteur Institute, Iran). Prior to the collection of the adipose tissue, mice were killed by cervical dislocation based an approval of the animal Ethics Committee (TMU.REC.1395.415) in Tarbiat Modares University (Tehran, Iran). During this study, we used standard protocols for the isolation of ADMSCs using collagenase enzymatic digestion. Briefly, the adipose tissue were minced and incubated with 0.1% Type-I collagenase (Invitrogen, USA) for 30 minutes at 37˚C. Collagenase activity was neutralized by addition of Dulbecco’s Modified Eagle’s Medium (DMEM, Gibco, USA) containing 20% heat inactivated fetal bovine serum (FBS, Gibco, USA). It was centrifuged at 1,700 rpm for 7 minutes. The supernatant was discarded and the pellet re-suspended in 1 ml of cell culture medium, consisting of DMEM, 20% FBS, and 1% penicillin/streptomycin (Gibco, USA). The cells were counted and seeded in a 75 cm^2^ flask with complete medium and incubated at 37˚C with 5% CO_2_ in humidified atmosphere. The medium was changed twice a week until 70-80% confluence as determined by microscope observation. Then they were harvested and expanded. All the experiments were performed using AD-MSCs at passages three.

### Adipose-derived-mesenchymal stem cells phenotyping

To analyze cell surface markers, AD-MSCs at passages 3 were harvested, washed with phosphate buffered saline (PBS), counted and 100 µl of the suspension incubated with monoclonal antibodies against defined markers CD29, CD34, CD45, CD90 and CD105 (all were purchased from BioLegend, USA) for 1 hour at 4˚C in the dark. The cells were fixed with 1% paraformaldehyde (Sigma-Aldrich, Germany) and analyzed using a FACSCanto II flowcytometer (BD Biosciences, USA) and FlowJo software.

### Adipogenic and osteogenic differentiation assay

Differentiation potential of AD-MSCs into adipogenic and osteogenic were assessed. Briefly, AD-MSCs were treated with osteogenic medium [10 mM betaglycerophosphate (Merck, UK), 50 mg/ml ascorbic acid- 2-phosphate (Sigma-Aldrich, Germany), and 100 nM dexamethasone (Sigma-Aldrich, Germany)] or adipogenic medium [250 nM dexamethasone (Sigma-Aldrich, Germany), 0.5 mM 3-isobutyl-1-methylxanthine (SigmaAldrich, Germany), 5 mM insulin (Sigma-Aldrich, Germany), and 100 mM indomethacin (Sigma-Aldrich, Germany)] for 3 weeks, with medium changes every 3-4 days. After 21 days, lipid droplets were visualized using Oil red O staining (ORO, Sigma-Aldrich, Germany) and to measure mineralization, osteogenic culture stained with Alizarin red S (ARS, Sigma-Aldrich, Germany).

### Culture of L929 cell and virus seed preparation

Murine L929 fibroblasts cells were a gift from Dr. Soudi (Tarbiat Modares University, Iran), propagated in DMEM containing 10% FBS, and 1% penicillin/streptomycin at 37˚C in a humidified 5% CO_2_ incubator. The cells were passaged at 80% confluency, and incubated at 37˚C with 5% CO_2_, 95% humidity.

The monolayers of L929 cells prepared in a 75 cm^2^ flask and infected with wild-type reovirus T3D [a generous gift from Dr. Shamsi-Shahrabadi (Iran University of Medical Sciences, Iran)] at multiplicities of infection (MOI) of 0.1 for virus seed preparation. The infected cells were incubated at 37˚C for 1 hour. Cells were washed twice with PBS and incubated in fresh medium at 37˚C. Virus stock was harvested when the virus cytopathic effects (CPE) become visible in more than 75% of the cells.

### Characterization of reovirus T3D

The plaque assay is one of the most efficient biological assays used for the quantification of reovirus T3D. This assay is based on the CPE, which was caused by active and replicable forms of reovirus in cell culture and introduced to plaque-forming units per milliliter of virus (PFU/ml). For this purpose, L929 cell in six-well culture plates overlaid with serial dilutions (10^-1^-10^-5^) of the reovirus T3D. After 1 hour, unabsorbed viruses were removed by washing twice with PBS. The cell monolayers were covered with a layer that contained 1% cell grade agar (Sigma, USA) in DMEM, 1% penicillin/streptomycin without serum. Plates were incubated at 37˚C for 4-5 days. The cells fixed using 3.7% formaldehyde for at least 2 h and plaques were then visualized by 1% crystal violet (CV) in 20% ethanol and dH_2_O.

For polyacrylamide gel electrophoresis (PAGE) and silver staining, double-stranded RNA of reovirus T3D was purified from the cells by RNA extraction solution (RiboEx, GeneAll, Korea). The genome of reovirus T3D was analyzed by electrophoresis on 12% polyacrylamide gels, and RNA segmented pattern confirmed by silver staining based on Laemmli protocol (17).

### Inoculation of adipose-derived-mesenchymal stem cells and L929 cell with different multiplicities of infection by reovirus T3D

AD-MSCs and L929 cell were cultured in DMEM that contained 10% FBS, 1% penicillin-streptomycin in 6 well plates for 24 hours. Subsequently, cells were washed twice with PBS, inoculated with a MOI of 10, 1, 0.1, 0.01 and 0.001 of reovirus T3D stock. After 1 hour of adsorption at 37˚C, the cells were washed twice with PBS and incubated at 37˚C in 1 ml FBS free DMEM supplemented with 1% penicillin-streptomycin.

Then, culture supernatants of cells harvested and analyzed for 50% cell culture infectious dose (CCID_50_) and quantitative real-time polymerase chain reaction (qRT-PCR) at the following time points: 1, 2, 3, 4, 5, 6, 7, 8, 12, 24, 48, 72 and 96 hours post-infection.

### Reovirus titration in adipose-derived-mesenchymal stem cells and L929 cell by CCID_50_ assay

AD-MSCs and L929 cell monolayer were prepared in a 48-well plate. Logarithmic dilutions (10^-1^-10^-10^) of each time point culture supernatants made in serum-free DMEM. The cells infected with each dilution, and the infected cells were examined for CPE presentation 72 hours post-infection. CPE results considered by comparing with positive (undiluted virus stock) and negative cell controls. Virus titers were calculated according to the method of Reed & Muench.

### Primer designing, amplification and sequencing of polymerase chain reaction product

The primer for reovirus T3D genomic *L3* gene segment (major capsid protein lambda 1) was designed by Lasergene. The primers for amplification of *L3* gene are

F: 5′-CGCGTCCTCAATTTTGGGTAAAC-3′

R: 5′-CCGCCGTCTTTTGGATATGAACTA-3′.

To confirm the specificity of the designed primers, a PCR reaction was performed with the following conditions: The final PCR reaction volume was 25 μl with forward and reverse primers concentration at 10 pmol/µL. The first round PCR starts at 95˚C for 2 minutes, followed by 35 cycles of 95˚C for 20 seconds, 61˚C for 40 seconds, 72˚C for 1 minute, with a final extension of 72˚C for 5 minutes with Applied Biosystems PCR platforms.

The 135 bp PCR product was subsequently evaluated and visualized by electrophoresis on 2% agarose gel alongside the 100 bp DNA ladder (DM2300 ExcelBand, Taiwan). PCR products were isolated with the QIAquick Gel Extraction Kit (Qiagen, Germany) and directly sequenced with an Applied Biosystems (ABI) 3130 genetic analyzer (Tehran University of Medical Sciences, Iran). The sequence was compared to the Gene Bank database using the BLAST databases available on National Center for Biotechnology Information (NCBI).

### Time point measurement of reovirus infectivity titers in adipose-derived-mesenchymal stem cells and L929 cell by real time quantitative polymerase chain reaction

A real-time PCR was developed to quantify reovirus T3D genomic RNA using the L3 gene segment with indicated primer sets in previous section. Absolute viral RNA load quantitation within culture supernatants of infected mouse AD-MSCs and L929 fibroblasts were used for the construction of a standard curve. Viral RNA was extracted from each time point culture supernatants using the High Pure Viral Nucleic Acid Kit (Roche, Germany) according to the manufacturer’s instructions. Extracted RNA was reverse transcribed into complementary DNA (cDNA) using cDNA synthesis kit (GeneAll, Korea), which included hexamer primers.

This assay was carried out on a serial logarithmic dilutions of virus positive control for each sample in order to construct the standard curves. Copy numbers for the standards were calculated based on Qiagen protocol (18). The reaction was carried out with EvaGreen/ Fluorescein master mix using Step One Plus Real-Time PCR System (Applied Biosystems, USA). A total volume of 20 µl amplification mixtures contained: 5X HOT FIREPol® EvaGreen® qPCR Mix Plus (ROX) 4 µl, forward and reverse primer (10 pmol/µL) 0.8µl, cDNA template 1 µl (225 ng/µl), nuclease-free water 14.2 µl. Reactions were run on a Step One Plus Real-Time PCR System. The cycle conditions were "holding stage 95˚C for 15 minutes; cycling stage 95˚C for 15 seconds and 61˚C for 20 seconds, 72˚C for 30 seconds for 40 cycles and a melt curve stage of 95˚C for 15 seconds, 70˚C for 1 minute and 95˚C for 15 seconds".

### Comparison the rate of adsorption and penetration in adipose-derived-mesenchymal stem cells and L929 cell

We demonstrated the penetration and adsorption rates in AD-MSCs and L929 cell with oncolytic reovirus. In order to obtain this ambition, cells were infected with MOI: 1 of reovirus. Extra unabsorbed virus was removed 1-1.5 hour post-infection. Then, infected cells were collected and the viral genome was extracted by High Pure Viral Nucleic Acid Kit (Roche, Germany) according to the manufacturer’s instructions. Synthesis of cDNA and Real-time PCR amplification was done similar to the previous section.

### Statistical analysis

Data analysis was done by REST program using Real Time PCR outputs. Standard curves for each sample were constructed by plotting Ct values versus the viral RNA copy number using the StepOne Software (Applied Biosystems).

All experiments were performed in triplicate and repeated three times. All data were analyzed by Excel 2016 and GraphPad Prism 7.04 (GraphPad Software, USA) and reported as mean ± standard deviation (SD).

## Results

### Characterization of adipose-derived-mesenchymal stem cells

**Fig 1:**
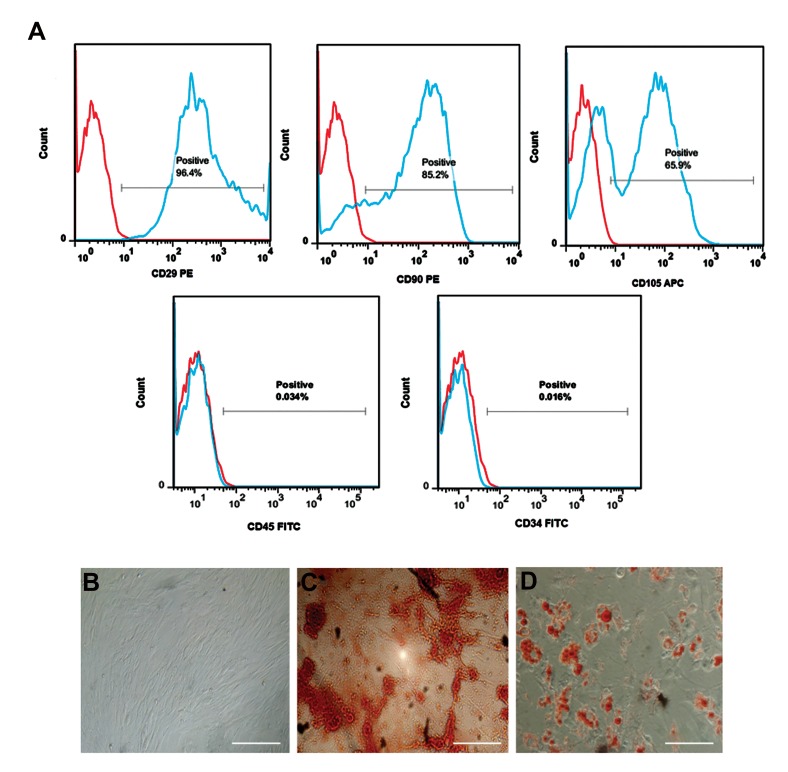
Characterization of adipose-derived-mesenchymal stem cells (AD-MSCs). **A.** Flow cytometry of AD-MSCs performed with monoclonal antibodies to detect cell surface markers. The expression of isotype controls is shown as red histograms, **B.** Fibroblast-like morphology of AD-MSCs at passage 3 in culture (scale bar: 100 µm), **C.** Matrix mineralization during osteogenesis of AD-MSCs was detected by Alizarin red S after 21 days of culture (scale bar: 20 µm), and **D.** Lipid droplets produced in AD-MSCs cultures after 21 days of adipogenesis, were stained by Oil red O (scale bar: 100 µm). The figure shows one representative results from three independent experiments.

Cell surface markers of AD-MSCs isolated from C57BL/6 mice at passage three were examined by flow cytometry. AD-MSCs showed low expression of CD34 and CD45 markers, but CD29, CD90 and CD105 markers were expressed at mean percentages of 96.4, 85.2 and 65.9%, respectively (Fig .1A). Fibroblastlike morphology of AD-MSCs at passage three are presented in Figure 1B. Adipogenic and osteogenic differentiation potential of AD-MSCs was confirmed by ORO and ARS staining as indicated in Figure 1C and 1D, respectively.

### Propagation of reovirus in L929 cell

We optimized the propagation conditions of reovirus by the sequential passage at MOI: 0.1 to reduce the rate of mutations. CPE has progressed to fully disrupt, three days after infection with reovirus T3D. Reovirus was released by the lysis of L929 infected cell and total cell lysate and medium was collected.

### Characterization of reovirus T3D

**Fig 2:**
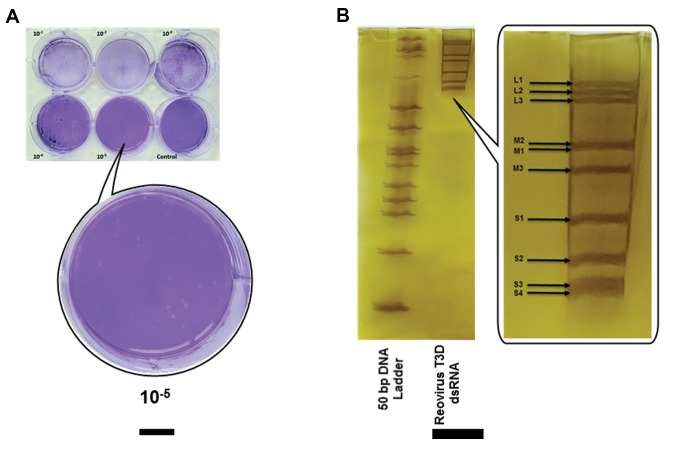
Characterization of reovirus T3D. **A.** Plaques formation by reovirus on monolayer L929 cell. About 50 plaques were counted for replicates of the 1×10^-5^ dilution, and the virus titer was 0.7×10^7^ PFU/ml. **B.** Electrophoretic migration pattern of dsRNA of reovirus T3D (3-3-2-2). RNA samples were analyzed by electrophoresis in a 12% PAGE gel and visualized by silver staining.

The visible plaques were formed within four to five days after reovirus inoculation (Fig .2A). The nominal titers of virus stocks were calculated according to the current microbiology protocol (19).

The viral dsRNA was purified and separated by electrophoresis on 12% polyacrylamide gel and RNA segmented pattern confirmed by silver staining as shown in Figure 2B. The result showed normal RNA migration pattern of reovirus T3D in polyacrylamide gel electrophoresis.

### Inoculation of reovirus in adipose-derivedmesenchymal stem cells and L929 cell

**Fig 3:**
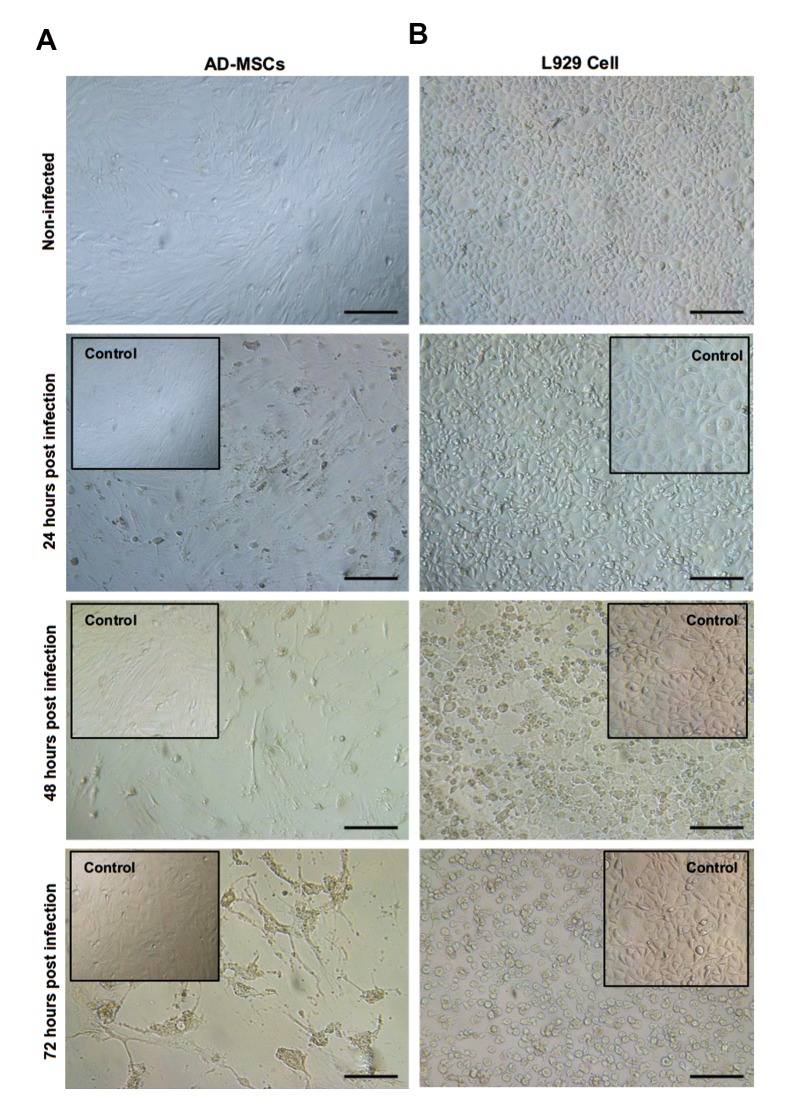
Phase contrast microscopy of confluent monolayer non-infected and infected AD-MSCs, L929 cell with MOI: 1, considering reovirus CPE in different time points. **A.** In AD-MSCs and **B.** In L929 cell (scale bars: 100 μm). AD-MSCs; Adipose-derived mesenchymal stem cells, MOI; Multiplicities of infection, and CPE; Cytopathic effect.

CPE in MOI: 1 of reovirus was obvious in both cells as shown in Figure 3 at deferent time points. At 24 hours post-infection, CPE was observed and completed at 72 hours post-infection.

### Determining the highest dilution of virus suspension in cell infectious dose using the 50% cell culture infectious dose assay

**Fig 4:**
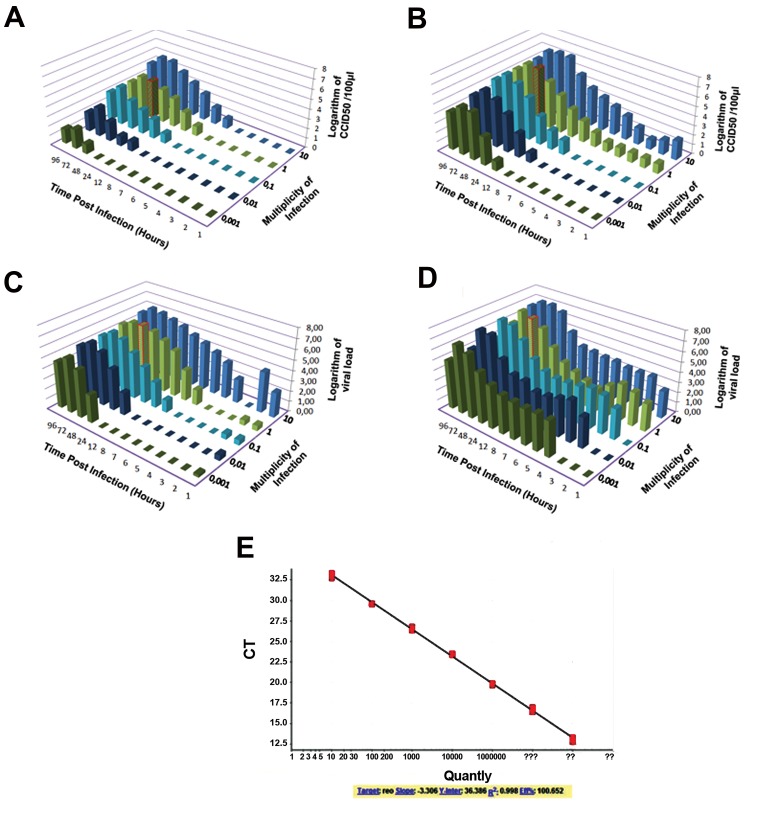
Kinetics of reovirus replication measured by CCID_50_ and qRT-PCR assay. **A.** Based on CCID_50_ results in mouse AD-MSCs, **B.** Based on CCID_50_ results in murine L929 fibroblasts cell. The results were expressed as logarithm of CCID_50_/100 μl. The growth curve of reovirus in AD-MSCs and L929 cell shown that, MOI: 1 might be optimal for virus production. In L929 cell, at MOI: 1 a regular rise in infectious virus titers was observed at 6 hours post-infection, reaching to its maximum level at 48 hours post-infection; but in AD-MSCs, at MOI: 1, virus progeny production was observed 7 hours post-infection, reaching to its maximum level at 48 hours post-infection. Data are expressed as the mean of three independent experiments, **C.** Based on qRT-PCR results in mouse AD-MSCs, **D.** Based on q RT-PCR results in murine L929 fibroblasts cell. The results were expressed as logarithm of copies/ml, and E. Ten-fold serial dilutions (10^1^-10^7^ copies/ml) of synthetic viral RNA standard were used to generate a standard curve. The growth curve of reovirus in AD-MSCs and L929 cell shown that, MOI: 1 might be optimal for virus production. In L929 cell, at MOI: 1 a regular rise in infectious viral load was observed at 5 hours post-infection, reaching to its maximum level at 48 hours post-infection; but in AD-MSCs, at MOI: 1, a regular rise in infectious viral load was observed 6 hours post-infection, reaching to its maximum level at 48 hours post-infection. Data are expressed as the mean of three independent experiments. CCID_50_; Cell culture infectious dose 50%, qRT-PCR; Quantitative real-time polymerase chain reaction, AD-MSCs; Adipose-derived mesenchymal stem cells, and MOI; Multiplicities of infection.

L929 cell and AD-MSCs were infected with different MOIs. Supernatants were collected in different time intervals [1, 2, 3, 4, 5, 6, 7, 8, 12, 24, 48, 72 and 96 hours post-infection] and then virus infection was determined by CCID_50_. As shown in Figure 4A, when AD-MSCs were infected with MOI: 10, the virus infectivity assay was positive 6 h post-infection. At MOI: 1, virus progeny production was observed 7 hours post-infection, reaching to its maximum level at 48 hours post-infection. In the current study, virus progeny production was initiated at the following MOI: 0.1 (t8), 0.01 (t12) and 0.001 (t48) and at 72 hours post-infection, the virus titer was reached a peak.

Infection of L929 cells with a higher MOI (MOI of 10 and 1) contain more residual infectious virus during adsorption and penetration and resulted in a productive infection earlier compared to the lower infectious virus titers as seen in Figure 4B. At MOI of 10 and 1 a regular rise in infectious virus titers were observed at 4 and 6 hours post-infection, reaching to its maximum level at 24 and 48 hours post-infection, respectively.

At MOI: 0.1 a rise in infectious virus titers was observed at 7 hours post-infection, reaching to its maximum level at 48 hours post-infection. Whereas for MOI of 0.01 and 0.001 progeny viruses was verified at 8-12 hours post-infection, reaching to its maximum level at 72 hours post-infection.

### Absolute viral RNA load quantitation

Agarose gel electrophoresis was used for separating of PCR product with detectable size of 135 bp (data not shown). The cDNA sequence analysis confirmed that the PCR products corresponded to the reovirus L3 gene segment (data not shown).

Amplification graph and melt curve analysis for each samples confirmed the specificity of the virus shedding. The viral RNA load (RNA logarithm of copies/ml), in each time point of AD-MSCs and L929 cell supernatants were calculated in comparison with standard curve (serial logarithmic dilutions of positive control) as illustrated in Figure 4C, D, and E, respectively. According to the result, Infection of both cells with different MOIs contain more residual infectious virus during adsorption and penetration and resulted in a productive infection earlier. In L929 cell, at MOI: 1 a regular rise in infectious viral load was observed at 5 hours postinfection, reaching to its maximum level at 48 hours post-infection; but in AD-MSCs, at MOI: 1, a regular rise in infectious viral load was observed 6 hours postinfection, reaching to its maximum level at 48 hours post-infection.

### The result of viral adsorption and penetration in adipose-derived-mesenchymal stem cells and L929 cell

**Fig 5:**
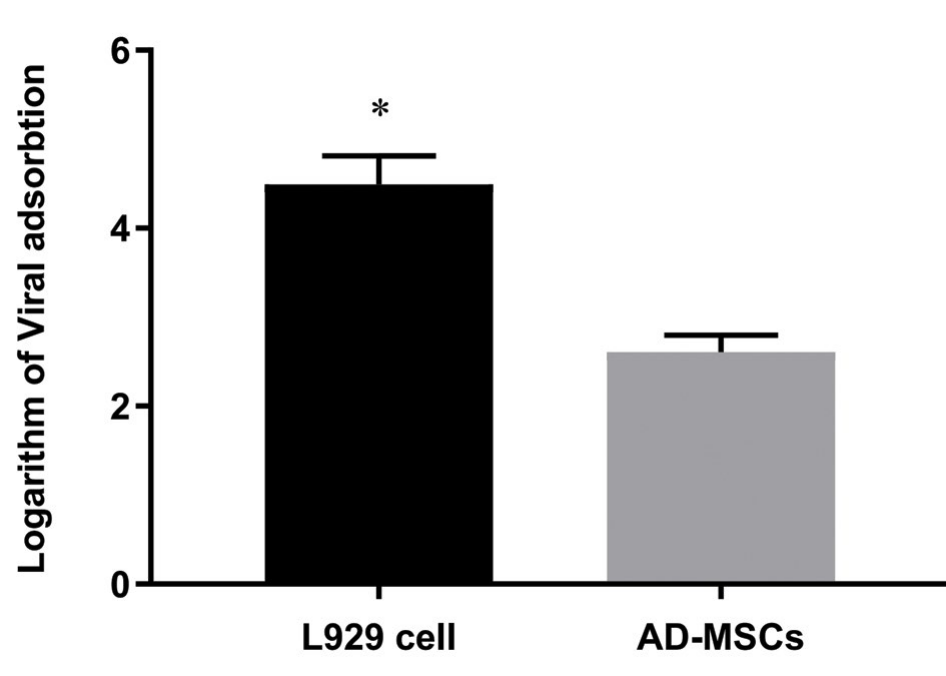
The rates of reovirus adsorption and penetration to AD-MSCs in comparison with L929 cell in MOI: 1. The results were expressed as logarithm of copies/ml. At this MOI, the virus adsorption and penetration in L929 cell is much more efficient than AD-MSCs. Data are expressed as the mean ± SD of three independent experiments. *; Indicated groups are significantly different from each other (P<0.05), AD-MSCs; Adiposederived mesenchymal stem cells, and MOI; Multiplicities of infection.

Based on real-time quantitative PCR result, no significant differences were observed in the rates of adsorption and penetration between different MOI (data not shown), but as demonstrated in Figure 5, the virus adsorption and penetration of MOI: 1 in L929 cell is much more efficient than AD-MSCs.

### Single cell cycle experiment using 50% cell culture infectious dose

**Fig 6:**
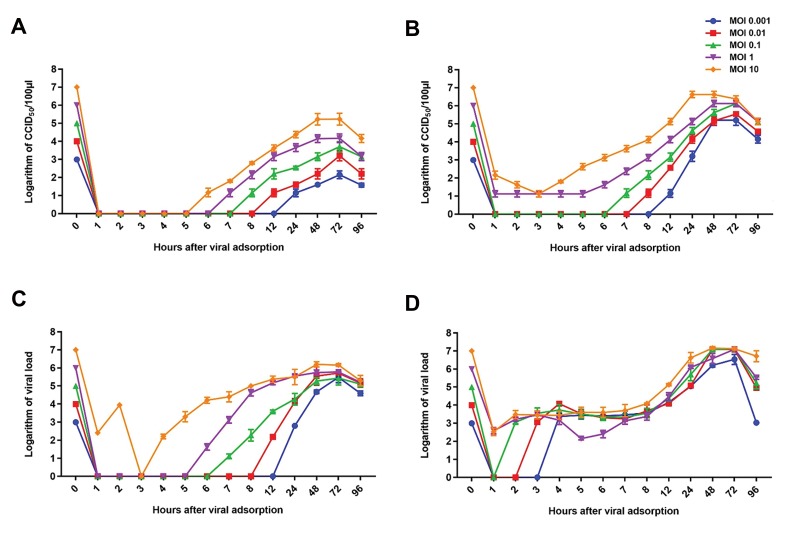
One-step growth curves of reovirus at different MOIs. **A.** Based on CCID_50_ results in AD-MSCs, **B.** Based on CCID_50_ results in L929 cell. The results were expressed as logarithm of CCID_50_/100 μl, **C.** Based on viral load results in AD-MSCs, **D.** Based on viral load results in L929 cell. The results were expressed as logarithm of copies/ml. The growth curve of reovirus by CCID_50_ and qRT-PCR in both cells showed that MOI: 1 might be optimal for virus production compared to higher and lower MOIs. Data are expressed as the mean ± SD of three independent experiments. MOI; Multiplicities of infection, CCID_50_; Cell culture infectious dose 50%, AD-MSCs; Adipose-derived mesenchymal stem cells, and qRT-PCR; Quantitative real-time polymerase chain reaction.

The results of a one-step growth experiment establish a number of important features about viral replication. According to Figure 6A, the result of reovirus one-step growth on AD-MSCs showed, at time points 5 (MOI: 10), 6 (MOI: 1), 7 (MOI: 0.1), 8 (MOI: 0.01) and 12 (MOI: 0.001) hours post-infection constitutes the eclipse period. Exponential growth of virus was started at 6, 7, 8, 12, 24 hours post-adsorption, in different MOI of 10, 1, 0.1, 0.01, 0.001 respectively. The quantity of infectious virus begins to increase and CPE was detected, marking the onset of the synthetic phase, and continued by assembly of new virus particles. Ultimately, viruses are released and the growth cycle enter the stationary and decline phases and further not supported to additional replication round.

According to Figure 6B, the result of reovirus one-step growth in L929 cell showed, at time points 3 (MOI: 10), 5 (MOI: 1), 6 (MOI: 0.1), 7 (MOI: 0.01) and 8 (MOI: 0.001) hours post-infection, constitutes the eclipse phase and viral nucleic acid uncoating from its protective shells. The exponential phase of reovirus infection in L929 cell was initiated at time points 4, 6, 7, 8 and 12 hours after adsorption with different MOIs of 10, 1, 0.1, 0.01 and 0.001, respectively; and the quantity of infectious virus begins to increase and CPE were detected.

## Discussion

OVs are able to infect different kinds of host cells (20). The progression process occurs at 2-4 hours or more than 1-2 days depending on MOI of virus and cell type. Wild-type oncolytic reovirus is an attractive anti-cancer agent for clinical testing (21). The OV therapy is not successful alone, because circulating antiviral antibodies in the blood neutralize the OVs before reaching to the target site; or perhaps macrophages recognize infected cells with OVs and kill them (22). The main problem in oncolytic virotherapy is delivery and interaction of OVs with the immune system (23). To resolve this issue, a novel approach has been suggested which is focused on the using of cell carriers (10). In the present study, in order to enhance the quantity and capabilities of the AD-MSCs for delivery of OVs, biosynthetic capacity of the AD-MSCs was assessed. The behavior of L929 cells as susceptible host cell line was studied in response to reovirus infection.

The behavior of the cells is different in response to viral infection. This variability among different infected cells can be attributed to cell properties, stages of the cell cycle (24), genetic severe heterogeneity of the virus population (25), or host cells resource differences (20). The replicability of an oncolytic reovirus is measured by its burst size, for further use in near future.

The quantitative description of the crucial steps in reovirus infection cycle has been presented in this study. We evaluated the preferential cytotoxicity and shedding of reovirus in AD-MSCs and compared them, with L929 cells as susceptible host cell line. For this purpose, MOI optimization and monitoring of reovirus shedding were done in the two mentioned target cells. The appropriate titer of virus for one step growth cycle was obtained by CCID_50_ and qRT-PCR.

According to the CCID_50_ data, the viral shedding was started from the early hours in infected L929 cells with the MOI of 10 and 1. This can be considered as false positive, because a lot of viral particles might have not been internalized or re-entered into the supernatant without infecting the cells and progeny production. As data represents, AD-MSCs at 48 hours post-infection with MOI: 1 had the highest titer of the virus shedding. The viruses entering stationary phase at the 72 hours post-infection. Then, the virus shedding decreased. In the higher MOI (MOI: 10), lysis of the cells occurred early after infection, and increased by the high titer of viruses. In this situation, the optimal rate of virus replication was low. According to Igase et al. (26), more than 50% of hang-up of cell growth was evidenced at MOI: 10 in the MGT cell lines. In the MOI lower than 1, the reproduction rate was low compared with MOI: 1, due to the lower titer of the virus. From a higher level of MOI to lower level, the production of progeny and approaching the pick value were delayed. Comparing the two cell lines, one log reduction in virus titer has been observed in ADMSCs in comparison with the L929 cell line. Jung et al. (27) reported that the final virus titer was closely linked to the input MOI and the host cell confluency at the time of infection. They reached a maximum virus titer when an MOI: 0.1 and the final host cell density of 1.0×10^6^ cells/ ml were used.

Based on qRT-PCR results, the viral shedding was started from the early hours in both infected cells with different MOIs. These can be considered as false positive, because a lot of viral particles might have not been internalized or re-entered into the supernatant without infecting the cells and progeny production. In MOI: 1, the ratio of virus to infected cells was optimum resulting in the highest level of virus replication similar to the results of CCID_50_ at 48 hours post-infection.

According to the one-step growth curve of reovirus, at MOI: 1 the viruses in both cells has the most regular replication cycle. The eclipse period of reovirus in L929 cell and AD-MSCs occurred 4-5 hours post infection. The growth curve of reovirus in AD-MSCs and L929 cell has demonstrated that the lower MOI might be ideal for high virus production compared to higher MOI as seen in the literature. Parallel finding was reported by Grande and Benavente (28) in chicken embryo fibroblast cells infected with avian reovirus S1133.

In both cells the maximum virus titer in MOI: 1 was reached at 48 hours post-infection, then stabilized and gradually decreased. The adsorption time, rise time and the time interval over which the cell produces virus are different in cell types. These are affected by the number of infected cells. This finding illustrates that the optimum titer depends on the virus-cell ratio rather than the concentration of virus and cells for progeny production.

The evaluation of the penetration and adsorption rates have shown no obvious difference between different MOIs. These rates absolutely depends on cell type and other environmental elements. The importance of cell source, MOI and the distribution of virus yields could reflect different numbers of adsorbed virus particles to distinct cells when they are treated with different MOIs. The average yield from the single cells does not change significantly, but intact virus production remains to be determined.

Taken together, the comprehensive range of virus yields from different cells, potentially reflects different factors such as genetic variation, and the cell type in the replication kinetics during the early stages of growth cycle.

## Conclusion

Based on the observed results, the cytopathic effect was seen in both cells, but one log reduction in virus titer and shedding in AD-MSCs was seen compared to L929 cell. According to their innate proliferation properties, ADMSCs can be susceptible but are less permissive to viral infection. The suitability of AD-MSCs as efficient carriers for wild-type oncolytic reovirus to target the cancer cells will be considered for further investigation.

These interpretations arise several questions about factors that influence virus–host interactions. We illustrated that the host cell resources capacity, virus MOI variation and burst size can affect strength of virus progeny production. A delay in adsorption and release of reoviruses in AD-MSCs could lead to the delivery of effective virus progeny at the right time per infected host as a carrier cell. This phenomenon needs further investigation for using infected AD-MSCs by oncolytic activity of reovirus in cancer therapy.
